# AGING-IN-COMMUNITY MODELS AND PROGRAMS: PROGRAM AND POLICY PLANNING STRATEGIES

**DOI:** 10.1093/geroni/igz038.1519

**Published:** 2019-11-08

**Authors:** Su-I Hou, Carrie Graham, Emily Greenfield

**Affiliations:** 1 The University of Central Florida, Orlando, Florida, United States; 2 University of California, Berkeley, California, United States; 3 Rutgers University, New Brunswick, New Jersey, United States

## Abstract

This symposium introduces key aging-in-community models and programs, with a particular focus on program and policy planning strategies. Villages are a new, consumer-directed model that aim to promote aging -in-community through a combination of facilitated social engagement, member-to-member support, volunteer opportunities, and collective bargaining for services. Dr. Graham from the University of California will share results from both a national survey of Village directors and a survey of village members, summarizing Village organizational development trends and members’ perceived impacts. Dr. Gilcksman from Philadelphia Corporation for Aging will share how older adults who do not participate in a Village create their own informal social and service network to maintain themselves and to accomplish the same goals as a Village, building community at the neighborhood level. Additionally, Dr. Hou from the University of Central Florida will discuss lessons learned on program planning strategies among older adults participating in three programs promoting aging-in-community: a university-based lifelong learning program (LLP), a county neighborhood lunch program (NLP), and a Florida Village program as a comparative case study. Finally, Dr. Glass from the University of North Carolina Wilmington will share the current trend of the new senior cohousing model, promises and challenges for older adults providing mutual support to each other as they age together. This symposium will further discuss strengths and weakness, and planning strategies of the various AIC models and programs.

